# Mechanism of spindle assembly regulated by the human oocyte microtubule organizing centre in human oocytes

**DOI:** 10.1002/ctm2.1222

**Published:** 2023-09-15

**Authors:** Tianyu Wu, Qing Sang, Lei Wang

**Affiliations:** ^1^ Institute of Pediatrics, Children's Hospital of Fudan University, State Key Laboratory of Genetic Engineering Institutes of Biomedical Sciences, Shanghai Key Laboratory of Medical Epigenetics, Fudan University Shanghai China

1

In female meiosis, the immature germinal vesicle (GV) oocyte is matured with two reductional divisions, which requires a meiotic spindle to segregate chromosomes sequentially. The abnormal spindle assembly of meiosis I leads to oocyte metaphase I (MI) arrest or chromosome abnormalities.^1^ Clinically, the meiotic spindle of human oocyte is often unstable, which induces chromosome segregation errors, and is a leading cause of infertility, miscarriage and congenital defects. However, the mechanism of spindle assembly in human oocytes was not understood for decades.^2^

The mechanism of spindle assembly was initially investigated in mitotic cells. During mitosis, opposite‐paired centrosomes are responsible for microtubule polymerization and bipolar spindle assembly. Centrosomes consist of a pair of centrioles surrounded by pericentriolar material (PCM) that is composed of multiple proteins required for microtubule localization or polymerization.^3^ Compared to mitotic cells, centrosomes are eliminated during oogenesis and absent from the meiotic spindle assembly of oocytes in most organisms including humans. In terms of the mechanism of spindle assembly in mouse oocytes that are well investigated previously, the function of canonical paired centrosomes is replaced by several acentriole microtubule organizing centres (aMTOCs) that only retain the PCM components. With the meiosis resumption, multiple aMTOCs are initiated to polymerize microtubules in the cytoplasm of mouse oocytes.^4^ The aMTOCs migrate to the nucleus and decondense on the nuclear envelope.^5^, ^6^ And then, the aMTOCs are stretched and fragmented along the nuclear envelope by dynein. After nuclear envelope breakdown (NEBD), kinesin superfamily protein 11 further fragments the aMTOCs to assemble the acentrosomal bipolar spindle (Figure 1).^5^ Therefore, the aMTOCs serve as the main regulator for mouse oocyte spindle assembly.

**FIGURE 1:**
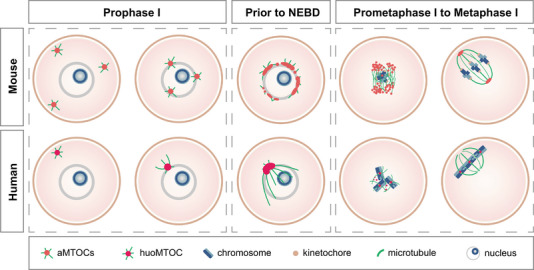
The distinct mechanisms of meiotic spindle assembly in mouse and human oocytes. The differences in the spindle assembly between mouse and human oocytes are described from prophase to metaphase in meiosis I. The spindle assembly and microtubule polymerization are achieved by acentriole microtubule organizing centres (aMTOCs) in mouse oocytes (top) but regulated by human oocyte microtubule organizing centre (huoMTOC) in human oocytes (bottom).

Different from mouse oocytes, the typical aMTOCs were not detected on the meiotic spindle of human oocytes for a long time,^2^ indicating a novel mechanism of acentrosomal spindle assembly. In the limited investigations about human oocytes, the spindle microtubules were observed emanating from chromosome aggregate after NEBD by immunofluorescence, and the spindle was assembled in a lengthy process without prominent aMTOCs.^7^ Although it has long been reported that efficient microtubule polymerization and stable spindle assembly are essential for female meiosis,^7^, ^8^ the mechanism of microtubule polymerization and spindle assembly in human oocytes was still not clear.

In the latest investigations, we have initially shed light on how the human oocyte polymerize microtubules and assemble spindle. In the live cell time‐lapse imaging, spindle microtubules were observed polymerizing from kinetochores in human oocytes, which is consistent with immunofluorescence imaging. Therefore, we assume that an unknown protein complex that is responsible for spindle microtubule polymerization could be functional on the kinetochores. To identify the complex, 86 centrosomal or microtubule‐related proteins were examined on over 2000 MI oocytes by immunofluorescence imaging. Four proteins (CCP110, CKAP5, DISC1 and TACC3) were identified on both kinetochores and microtubules, which indicates that they could play roles in microtubule polymerization or spindle assembly.^9^ Trace to its source, we test the localization of the proteins in human GV oocytes, and surprisingly, discovered a novel subcellular structure that is surrounded by abundant microtubules. According to its ability of microtubule polymerization, we termed the novel subcellular structure a “human oocyte microtubule organizing centre (huoMTOC)”.

With the live cell time‐lapse microscopy of human oocytes, the mechanisms of huoMTOC‐regulated spindle assembly were observed in detail. The huoMTOC was recruited to the nucleus prior to NEBD and then fragmented into several pieces. The fragmented huoMTOC was then relocated to kinetochores to polymerize spindle microtubules (Figure 1). The disruption of huoMTOC by core proteins downregulation or laser ablation has blocked spindle microtubule polymerization and stopped spindle assembly.^9^ Therefore, the mechanism of spindle assembly dominated by huoMTOC is indispensable for human oocyte meiosis.

The importance of huoMTOC was not only shown in biological experiments but also play a key role in a clinical disease named oocyte maturation arrest. Whole exon sequencing screening in over 1400 patients with oocyte MI arrest revealed that two patients carried compound variants of *TACC3* that code a core protein member of huoMTOC.^9^ To figure out the reason for MI arrest of oocytes, the oocytes from patients were examined by immunofluorescence and found that not only the huoMTOC was unobservable in GV oocytes but also the spindle was absent in MI oocytes. Therefore, we have shown that the abnormal huoMTOC could induce clinical human oocyte maturation arrest.

In conclusion, although the mechanisms of spindle assembly regulated by huoMTOC have been investigated in both physiology and pathology, additional molecular players and mechanisms that are involved in the formation and migration of huoMTOC, polymerization of spindle microtubule, organization of spindle pole and stabilization of bipolar spindle are largely unknown. These need further exploration and discovery in the future.

## CONFLICT OF INTEREST STATEMENT

The authors declare no conflict of interest.
